# Early prediction of cerebral-cardiac syndrome after ischemic stroke: the PANSCAN scale

**DOI:** 10.1186/s12883-020-01833-x

**Published:** 2020-07-08

**Authors:** Haijuan Lian, Xiaomeng Xu, Xuhui Shen, Jinhua Chen, Dandan Mao, Yan Zhao, Meiqi Yao

**Affiliations:** 1 Hangzhou Medical College, Hangzhou, 310053 China; 2 The Second Affiliated Hospital, School of Medicine, Zhejiang University, 88 Jiefang Road, Hangzhou, 310009 China; 3 Medicine & Nursing Science School, Huzhou University, 1 Xueshi Road, Huzhou, 313000 China; 4 Ruijin Hospital, Shanghai Jiaotong University School of Medicine, 197 Ruijin Second Road, Shanghai, 200025 China

**Keywords:** Stroke, Cerebral cardiac syndrome, Risk prediction model

## Abstract

**Background:**

Cerebral-cardiac syndrome, newly developed cardiac damage manifestations subsequent to cerebral injuries, is a common complication of stroke and leads to increased morbidity and mortality. The current study is aimed to develop a risk prediction scale to stratify high-risk population of CCS among ischemic stroke patients.

**Methods:**

The study included 410 cases from four tertiary medical centers from June 2018 to April 2019. The risk prediction model was established via logistic regression from the derivation cohort including 250 cases admitted between June 2018 and December 2018. Another 160 cases admitted from January 2019 to April 2019 were included as the validation cohort for external validation. The performance of the model was determined by the area under curve of the receiver operating characteristic curve. A rating scale was developed based on the magnitude of the logistic regression coefficient.

**Results:**

The prevalence of CCS was 55.2% in our study. The predictive model derived from the derivation cohort showed good calibration by Hosmer-Lemeshow test (*P* = 0.492), and showed sensitivity of 0.935, specificity of 0.720, and Youden index of 0.655. The C-statistic for derivation and validation cohort were 0.888 and 0.813, respectively. Our PANSCAN score (0 to 10 points) was then established, which consists of the following independent risk factors: PT(12 s–14 s = 0; otherwise = 1), APTT(30s–45s = 0, otherwise = 1), Neutrophils(50–70% = 0; otherwise = 1), Sex(female = 1), Carotid artery stenosis(normal or mild = 0; moderate to severe = 2), Age(≥65 years = 1), NIHSS score(1 to 4 = 2; ≥5 = 3). Patients scored 3 or more points were stratified as high risk.

**Conclusion:**

The risk prediction model showed satisfactory prediction effects. The PANSCAN scale provides convenient reference for preventative treatment and early management for high-risk patients.

**Trial registration:**

The study was retrospectively registered in *Chinese Trial Registry*. The date of registration is April 17, 2019. Trial registration number: ChiCTR1900022587.

## Background

The interaction between the brain and the heart was recognized as early as the 1940s, when Byer and colleagues first reported abnormal electrocardiography (ECG) findings in six patients with heterogenous acute cerebral diseases including hypertensive encephalopathy and cerebrovascular disease (CVD) in 1947 [1]. Later on, abnormal ECG patterns in CVD were concluded by George Brunch and colleagues in 17 patients diagnosed with acute cerebrovascular accidents, among which 14 were hemorrhagic cases diagnosed by lumbar puncture, and three were unclassified cerebrovascular accident due to technique limitation at that time [2].

In the modern age, with decades of progress in medical science and technology, accumulating evidence further acknowledges the significance of brain-heart connection, which is now referred to as neurocardiology, [3] and the cardiac dysfunctions secondary to cerebral injury is now named as brain-heart syndrome or cerebral cardiac syndrome (CCS) [3, 4]. As previous study suggested that CCS was more common in CVD patients than in other neurological diseases, [5] recent studies further showed that CCS can occurred in 25–75% stroke patients, depending on different study design and examine methods [3, 6, 7]. CCS significantly affects prognosis, morbidity and mortality of stroke, and accounts for the second leading cause of death after cerebrovascular disease [3]. In one of the most severe cases, CVD will be complicated with acute myocardial infarction, which has no clear recommendation for ideal management while has an reported incidence of 0.52% within 24 h after CVD and 12.7% among geriatric patients within 72 h [8]. Therefore, the early detection and management of CCS is particularly important. However, despite the accumulating evidence from domestic and international studies, the underlying mechanism and pathogenesis of CCS are not fully understood and the risk factors for CCS are still controversial. In order to facilitate early stratification of high-risk CCS patients, our study was aimed to detect possible risk factors and to establish a predictive model for clinical practice.

The current study is aimed to identify potential risk factors for CCS after ischemic stroke, so patients with history of previous heart disease were excluded from the study to avoid confounders, and cardioembolic stroke was therefore excluded as well. As a result, the study is focused on the identification of independent predictors of CCS subsequent to non-cardiogenic ischemic stroke and the establishment of a risk prediction scale to stratify high-risk patients for early management in clinical practice.

## Methods

### Patients

The patients were included from four tertiary medical centers (the Second Affiliated Hospital of Zhejiang University School of Medicine, Ruijin Hospital Affiliated to Shanghai Jiaotong University School of Medicine, Hangzhou First People’s Hospital and Huzhou Central Hospital). For the derivation cohort, prospectively collected medical records of patients admitted from June 2018 to December 2018 were retrospectively investigated, and as a result a total of 250 cases were included. The data acquired from the derivation cohort was used to decide the sample size of the validation cohort with events per variable (EPV) method as reported previously [9]. Based on the analysis of the derivation cohort, the incidence of CCS was 55.2%, and after univariate and multivariate analysis, seven variables entered the predictive model. Therefore, according to the EPV method and considering 15% cases for incomplete data, sample size of the validation cohort was determined as: *N* = 7 × 10÷0.552 × 1.15 = 146. Eventually, 160 patients consecutively admitted between January 2019 and April 2019 were enrolled prospectively to formed the validation cohort.

Patients were included if aged ≥18 years old and diagnosed with acute stroke within 7 days of symptom onset. The diagnosis of stroke was based on persistent neurological deficits and confirmation by computed tomography (CT) and/or magnetic resonance imaging (MRI).

Exclusion criteria were as follows: (1) CT showed intracranial hemorrhage or subarachnoid hemorrhage; (2) incomplete data for myocardial zymogram, echocardiograph, and/or electrocardiogram (ECG) data within 48 h after admission; (3) history of cardiac disease before admission; (4) cardioembolic stroke subtype; (5) concurrent malignant neoplasm, severe liver, kidney or cardiopulmonary disorder.

### Diagnostic criteria of CCS

CCS was diagnosed according to the following criteria [10]: (1) no previous history of heart disease; (2) clear diagnosis of acute ischemic stroke; (3) any secondary cardiac damage manifestations. The manifestation includes: (1) ECG changes, including ST segment elevation or depression, T wave changes and QT prolongation [11–13]; (2) cardiac dysfunctions in echocardiography such as left ventricular diastolic dysfunction, [14] abnormal ventricular wall motion, [15] and decreased left ventricular ejection fraction [16]; (3) elevated peripheral cardiac markers by laboratory tests including troponin [17] and B-type natriuretic peptide [18].

### Statistical analysis

Continuous data was expressed as mean ± standard deviation (SD) and analyzed with *t* test if normally distributed, otherwise the data was expressed by the median and interquartile ranges, and Mann–Whitney U test was used. Categorical data was described by frequencies and percentage, and the *χ*^*2*^ test or Fisher’s exact tests was used when appropriate. Univariate analysis was performed to detect potential association between CCS and the following variables respectively: age, sex, subtype of stroke, vital signs on admission (temperature and mean arterial pressure), Glasgow Coma Scale (GCS), National Institutes of Health Stroke Scale (NIHSS) Score, medical history (hypertension, diabetes, previous stroke, smoking and drinking), laboratory tests, including white blood cell count, neutrophil, prothrombin time (PT), activated partial thromboplastin time (APTT), fibrinogen, D-Dimer, glutamic-pyruvic transaminase, serum potassium, glucose, urea nitrogen, creatinine, triglycerides and low-density lipoprotein, and echocardiography findings (intima-media thickness, carotid stenosis, and left ventricular ejection fraction). Carotid stenosis was classified according to NASCET criteria: < 30% is classified as mild, 30–69% is moderate, and > =70% is severe stenosis [19]. Data from the derivation cohort were entered into logistic regression to identify independent predictors and to develop the predictive model if the univariate analysis showed significant association (*P* < 0.05). The calibration were tested by Hosmer-Lemeshow (HL) goodness-of-fit test, and the discriminative ability was evaluated by C statistics in the derivation cohort and the validation cohort respectively. The rating scale was established with the magnitude of the logistic regression coefficient, and the cut-off valued was determined with receiver operating characteristic curve (ROC) curve. Statistical analysis was performed using SPSS 25.0 (IBM, Armonk, NY).

## Results

**Fig. 1:**
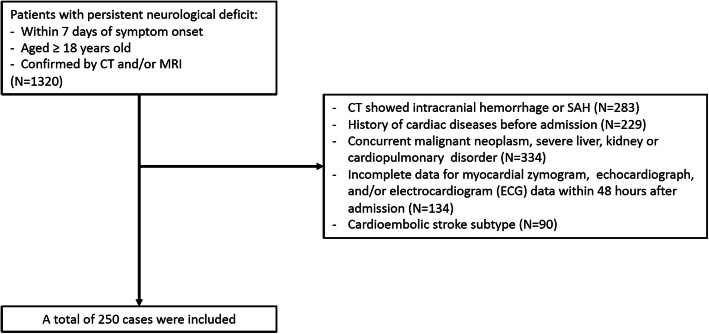
Flow chart of patient inclusion

**Fig. 2:**
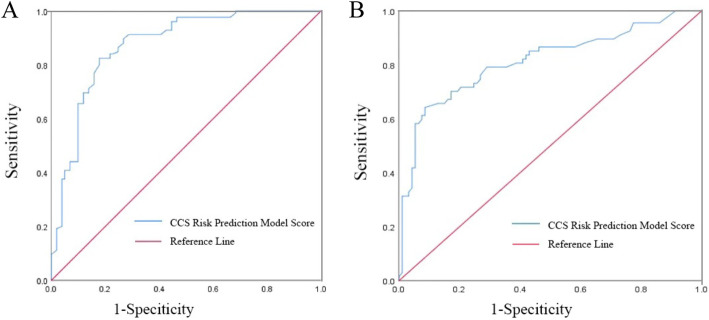
ROC curves of derivation cohort (Panel **a**) and validation cohort (Panel **b**)

Between June 2018 and December 2018, a total of 250 ischemic stroke patients were included as the derivation cohort **(**Fig. 1**)**, and among them 138 cases of CCS occurred, with an prevalence of 55.2%. Baseline characteristics was presented in Table 1. Univariate analysis showed that the following variables were significantly associated with the occurrence of CCS: age, sex, NIHSS, Neutrophil, PT, APTT, D-dimer, Carotid Stenosis and LVEF (Table 1). These variables were entered into multivariate logistic regression analysis, and seven independent predictors of CCS were identified: age, sex, NIHSS, Neutrophil, PT, APTT, Carotid Stenosis (Table 2). The HL test showed good calibration (*P* = 0.492). The discriminant validity of the model was evaluated by the area under the ROC curve (AUC), and finally AUC = 0.888, with sensitivity of 0.935, specificity of 0.720, and Youden index of 0.655 (Fig. 2. panel a).

**Table 1:** Baseline Characteristics of Patients in the Derivation Cohort (*n* = 250)

Characteristics		no CCS, *n* = 112	CCS, *n* = 138	*P*-value
Age, n (%)	< 65	76 (67.9)	50 (36.2)	< 0.001*
	≥65	36 (32.1)	88 (63.8)	
Sex, n (%)	Male	70 (62.5)	56 (40.6)	0.001*
	Female	42 (37.5)	82 (59.4)	
Stroke location, n (%)	Brain stem	18 (16.1)	30 (21.7)	0.155
	Others	94 (83.9)	98 (71.0)	
Temperature(°C), n (%)	<37.5	98 (87.5)	118 (85.5)	0.713
	≥37.5	14 (12.5)	20 (14.5)	
Mean Arterial Pressure (mmHg), n (%)	Normal(<105)	44 (39.3)	60 (43.5)	0.521
	Elevated(≥105)	68 (60.7)	78 (56.5)	
GCS Score, n (%)	Conscious(15)	108 (96.4)	120 (87.0)	0.027*
	Mild(12 ~ 14)	2 (1.8)	12 (8.7)	
	Moderate(9 ~ 11)	0 (0.0)	4 (2.9)	
	Coma(≤8)	2 (1.8)	2 (1.4)	
NIHSS Score, n (%)	0	54 (48.2)	20 (14.5)	< 0.001*
	1 ~ 4	44 (39.3)	60 (43.5)	
	> 4	14 (12.5)	58 (42.0)	
Hypertension, n (%)	No	32 (28.6)	58 (42.0)	0.34
	Yes	80 (71.4)	80 (58.0)	
Diabetes, n (%)	No	82 (74.5)	96 (70.6)	0.567
	Yes	28 (25.5)	40 (29.4)	
Stroke Times, n (%)	1 times	96 (85.7)	110 (79.7)	0.245
	> 1 times	16 (14.3)	28 (20.3)	
Smoking history, n (%)	No	104 (92.9)	122 (88.4)	0.284
	Yes	8 (7.1)	16 (11.6)	
Drinking history, n (%)	No	72 (64.3)	98 (71.0)	0.277
	Yes	40 (35.7)	40 (29.0)	
WBC(10^9/L), median (IQR)		6.30 (5.60, 8.18)	6.75 (5.43, 8.90)	0.497
Neutrophil(%), median (IQR)		61.3 (56.1, 66.1)	66.0 (59.6, 76.0)	< 0.01*
PT(s), median (IQR)		13.0 (12.3, 13.9)	13.6 (12.8, 15.1)	0.012*
APTT(s), median (IQR)		36.6 (34.4, 38.3)	37.2 (34.4, 42.0)	0.031*
Fibrinogen(g/L), median (IQR)		3.29 (2.80, 3.83)	2.96 (2.51, 3.80)	0.022*
D-Dimer(< 500 μg/l FEU), median (IQR)		390.0 (300.0, 630.0)	490.0 (300.0, 1130.0)	0.023*
GPT(U/L), median (IQR)		18.0 (13.0,29.0)	17.0 (13.0,27.0)	0.609
GOT(U/L), median (IQR)		22.0 (19.0,27..8)	23.0 (18.0,28.5)	0.813
Serum Potassium, median (mmol/l)(IQR)		3.72 (3.48,3.89)	3.79 (3.37,3.95)	0.997
Blood Glucose, median (mmol/l) (IQR)		5.22 (4.75,6.41)	5.21 (4.66,7.27)	0.760
BUN (mmol/l), median (IQR)		4.59 (3.67,5.61)	5.00 (4.15,6.14)	0.037*
Serum creatinine (mmol/l),median (IQR)		58.0 (49.3,70.8)	59.0 (47.8,71.5)	0.784
Triglycerides (mmol/l), median (IQR)		1.31 (0.98,1.69)	1.20 (0.87,1.57)	0.098
LDL (mmol/l), median (IQR)		2.28 (1.77,2.97)	2.10 (1.48,2.55)	0.010
IMT, n (%)	Normal	35 (34.7)	42 (34.1)	0.483
	Thicken	66 (65.3)	81 (65.9)	
Carotid Stenosis, n (%)	Normal	56 (55.4)	42 (33.6)	< 0.001*
	Mild	36 (35.7)	47 (37.6)	
	Moderate to Severe	9 (8.9)	36 (28.8)	
LVEF(%), n (%)	< 50	2 (2.2)	6 (5.0)	0.014*
	50 ~ 55	0 (0.0)	3 (2.5)	
	55 ~ 60	14 (15.1)	27 (22.3)	
	≥60	77 (82.8)	85 (70.2)	

^*^indicated *P* < 0.05

*Abbreviations*: *GCS* Glasgow coma scale, *NIHSS* National Institutes of Health Stroke Scale, *WBC* White blood cell, *GPT* Glutamic Pyruvic Transaminase, *GOT* Glutamic-Oxalacetic Transaminease, *BUN* Blood Urea Nitrogen, *LDL* Low Density Lipoprotein, *IMT* Intima-Media Thickness, *LVEF* Left Ventricular Ejection Fraction, *IQR* interquartile range, *FEU* Fibrinogen Equivalent Units

**Table 2:** Logistic multivariate regression analysis of CCS(*n* = 250)

Items	Category	Regression Coefficient	SD	Wald	*P- value*	*OR*	95% CI
Age	< 65	Reference	0.368	6.993	0.008	2.646	(1.286,5.442)
	≥65	0.973					
Sex	Male	Reference	0.367	5.577	0.018	2.38	(1.159,4.886)
	Female	0.867					
NIHSS Score	0	Reference		28.127			
	1 to 4	1.546	0.463	11.133	0.001	4.692	(1.892,11.633)
	≥5	2.76	0.521	28.031	< 0.01	15.797	(5.687,43.881)
Neutrophil	Normal (50–70%)	Reference	0.393	8.231	0.004	3.089	(1.429,6.673)
	<50% OR ≥ 70%	1.128					
PT	< 14 s	Reference	0.368	8.442	0.004	2.915	(1.416,5.998)
	≥14 s	1.07					
APTT	< 45 s	Reference	0.45	10.594	0.001	4.33	(1.791,10.465)
	≥45 s	1.466					
Carotid Stenosis	Normal	Reference		9.451			
	Mild	0.423	0.404	1.095	0.295	1.527	(0.691,3.373)
	Moderate to Severe	1.69	0.55	9.438	0.002	5.417	(1.843,15.919)

The model was validated with the external validation cohort including patients admitted from January 2019 to April 2019 (Table 3). Among the 160 cases, 67 CCS actually occurred, whereas 93 cases were predicted by model (Table 4). The results showed that the CCS prediction probability of the model was in good agreement with the actual incidence, and the difference was not significant(*P* = 0.067). The AUC of this model was 0.813, with the sensitivity of 0.642, the specificity of 0.914. (Fig. 2. panel b) The validation results showed the accuracy of 68.75%. suggesting that the CCS risk prediction model established in this study has good discriminating ability.

**Table 3:** Comparison of Risk Factors between Derivation and Validation Groups

	Derivation Group	Validation Group	*P*
n	250	160	
Age, n (%)			
< 65	126 (50.4%)	95 (59.4%)	0.085
≥ 65	124 (49.6%)	65 (40.6%)	
Sex, n (%)			
Male	126 (50.4%)	83 (51.9%)	0.840
Female	124 (49.6%)	77 (48.1%)	
NIHSS Score, n (%)			
0	74 (29.6%)	60 (37.5%)	0.070
1 ~ 4	104 (41.6%)	49 (30.6%)	
> 4	72 (28.8%)	51 (31.9%)	
Neutrophil(%), median (IQR)			
	150 (60%)	93 (58.1%)	0.757
	100 (40%)	67 (41.9%)	
PT(s), median (IQR)			
	136 (54.4%)	89 (55.6%)	0.839
	114 (45.6%)	71 (44.4%)	
APTT(s), median (IQR)			
	198 (79.2%)	115 (71.9%)	0.096
	52 (20.8%)	45 (28.1%)	
Carotid Stenosis, n (%)			
Normal	98 (43.4%)	79 (49.4%)	0.503
Mild	83 (36.7%)	52 (32.5%)	
Moderate to Severe	45 (19.9%)	29 (18.1%)	

*P*<0.05

**Table 4:** Model Validation (*n* = 160)

Model prediction	CCS actually occurred		Total
	No	Yes	
No	63	16	79
Yes	30	51	81
Total	93	67	160

Kappa value = 0.426

*p < 0.001*

Based on the logistic regression coefficients of the validated model, the PANSCAN scale was developed, with its items and corresponding scores presented in Table 5. The cut-off value was determined with ROC curve and Youden index. When Youden index reached the maximum, the cut-off value was 3 points. As a result, ischemic stroke patients with a PANSCAN score of 3 or more points were identified as high risk individuals.

**Table 5:** The PANSCAN Scale

Items	Category	Points
Age	< 65	0
	≥65	1
Sex	Male	0
	Female	1
NIHSS Score	0	0
	1 to 4	2
	≥5	3
Neutrophil	Normal (50–70%)	0
	< 50% OR ≥ 70%	1
PT	< 14 s	0
	≥14 s	1
APTT	< 45 s	0
	≥45 s	1
Carotid Stenosis	Normal	0
	Mild	0
	Moderate to Severe	2

## Discussion

CCS is a common acute complication of acute stroke, especially within 3 days of stroke onset [20]. In this study, the incidence of CCS was 55.2%. The cases included in this study were mainly from the Second Affiliated Hospital of Zhejiang University School of Medicine, Ruijin Hospital affiliated to Shanghai Jiaotong University School of Medicine, Hangzhou First People’s Hospital and Huzhou Central Hospital. The data were complete and credible. Although the current study was not extended to the prognosis or mortality, previous studies demonstrated that the development of CCS subsequent to stroke resulted in significant increase in hospitalization costs and prolonged hospital stay, [21] and severe cases can lead to worsened functional outcome and even death [3, 22]. Therefore, early identification and prevention of CCS were important in stroke patients.

In this study, we proved that that age, sex, NIHSS score, neutrophil, PT, APTT, and carotid stenosis were independent risk factors for CCS in stroke patients. Most of these findings were consistent with previous studies: higher age, female and coagulation were well-established risk factors for cardiac and cardiovascular events [6]. In addition, we found that carotid stenosis was also identified as an independent predictor for CCS. This finding is consistent with the previous publication by Gaia Sirimarco, which demonstrated that the presence of carotid stenosis is independently associated with increased risk of future coronary artery events and is a marker of disease severity [23]. Theoretically, carotid stenosis indicates the overall atherosclerosis level of vascular and can therefore suggest potential cardiovascular risk [24]. Pathological changes of carotid artery are widely adopted as surrogate for predictive risk factors for cardiovascular disease, [25] although evidence was conflicting. Previous studies demonstrated that intimal medial thickness and plaque prevalence are correlated with increased risk of cardiovascular disease, [26, 27] whereas recent study found that carotid plaque length can be a better predictor [28]. In our study, we found carotid stenosis was independently associated with CCS, which can be explained by the hypothesis that carotid stenosis represents long term accumulative exposure of cardiovascular risk factors [29].

Furthermore, we found that higher NIHSS score and elevated neutrophil counts also added to the risk for CCS. One possible explanation is the catecholamine surge hypothesis, the most widely accepted theory for the development of CCS [3]. According to the hypothesis, sudden and severe cerebral attack causes abnormal activation of autonomic nervous system that leads to catecholamine surge and results in cardiac dysfunction [3]. In support of this hypothesis, previous investigators showed that increased stroke severity was related to impaired cardiac autonomic modulation, [30] and was associated with higher cardiac mortality after stroke [31]. Consistently, our study identified that NIHSS, a direct index of stroke severity, was an independent predictor of CCS. Similarly, neutrophil is also a marker of stroke severity, [32] and is related to increased risk of new cardiovascular events, [33] as previous studies demonstrated.

This study included a large multicenter sample of Chinese population for analysis. Based on the results of univariate analysis and logistic multi-factor regression analysis in the derivation cohort and verification in the validation cohort, a risk prediction scale of CCS was established. It is suitable for the Chinese population to predict the occurrence of CCS. The model can be further promoted in clinical practice. The predictive model can contribute to clinical assessments for CCS risk, as well as health education, lifestyle interventions, and to improve patient compliance and satisfaction.

The major limitation is that based on the design of our study, patients with previous medical history of cardiac disorder and cardio-embolic stroke subtype was not included. However, in this population, attention on cardiac problems was routinely given at the first place, so the aim of the study is to stratify high-risk population without previous known heart diseases, among whom attention on cardiac conditions is often neglected. Another flaw is that we defined pre-admission history of cardiac disorder according to medical history of patients. Optimally, baseline cardiac evaluations should be accomplished immediately after stroke onset to exclude previous cardiac problems, but it is difficult to achieve in real world practice, and in our study, we included patients within 7 days after stoke. In this case, we reviewed patients’ past medical history and previous medical record as baseline cardiac conditions to minimize potential bias. Finally, our study only included Chinese population, so the generalization of our finding to other races and ethnicities was not validated in our study. Studies including larger sample size and other races and ethnicities are needed for further validation and generalization of the scale.

The PANSCAN scale established in this study is simple and feasible for clinical reference. In the future studies, prediction software can be developed to achieve intelligent and accurate warning of CCS, which will provide a reliable reference for clinical decision-making, and bring great convenience. Therefore, the results of this study have a good application value and prospects.

## Conclusion

This study used logistic regression analysis to screen the risk factors affecting the incidence of CCS, and to construct a scientific and effective risk prediction scale. This provides a practical method for objectively quantifying the risk of developing CCS in stroke patients. The scale shows practical clinical significance and convenience to prevent the occurrence of CCS and to improve the clinical prognosis of stroke。.

## Data Availability

The datasets used and/or analyzed during the current study are available from the corresponding author on reasonable request.
